# Predicted skeletal muscle index as a scalable marker for sarcopenia risk and mortality in older Chinese adults

**DOI:** 10.3389/fmed.2025.1640410

**Published:** 2025-08-20

**Authors:** Ya Liu, Yingsong Qi, Huanhuan Wang, Xiaojing Zhao, Min Zhang, Liangyan Ma, Lan Huang, Qiaorong Dong

**Affiliations:** ^1^Department of Nephrology, Affiliated Hospital of Chengde Medical University, Chengde, Hebei, China; ^2^Department of Cardiothoracic Surgery, Chengde Central Hospital, Chengde, Hebei, China

**Keywords:** sarcopenia, predicted skeletal muscle index, mortality, older adults, blood-based biomarker

## Abstract

**Background and aims:**

Sarcopenia, characterized by age-related loss of muscle mass and function, increases adverse outcomes in older adults. The predicted skeletal muscle mass index (pSMI), derived from serum creatinine and cystatin C, may serve as a practical biomarker. This study evaluated pSMI’s ability to predict sarcopenia and mortality in older Chinese adults.

**Methods:**

We analyzed 5,982 adults aged ≥60 years from the 2015 China Health and Retirement Longitudinal Study (CHARLS). pSMI was calculated using a sex-specific formula and categorized into quartiles. Sarcopenia was defined per Asian Working Group for Sarcopenia 2019 criteria. All-cause mortality was tracked over 5 years (2015–2020). Logistic regression assessed pSMI’s association with sarcopenia, and Cox models evaluated mortality risk, adjusting for demographics, behaviors, comorbidities, and inflammatory markers.

**Results:**

Sarcopenia prevalence decreased from 55 to 67% in the lowest pSMI quartile (Q1) to 0.4% in the highest (Q4). Higher pSMI reduced sarcopenia odds by 97–99% in Q3–Q4 vs. Q1 (*p* < 0.001). Five-year mortality fell from 23.4% (men) and 13.6% (women) in Q1 to 9.0 and 3.9% in Q4. Adjusted hazard ratios for mortality in Q4 vs. Q1 were 0.47 (95% CI: 0.32–0.70, men) and 0.38 (95% CI: 0.22–0.65, women).

**Conclusion:**

pSMI strongly predicts sarcopenia and mortality in older Chinese adults. To our knowledge, this represents the first large-scale validation of pSMI’s prognostic value for mortality risk independent of baseline sarcopenia in a nationally representative older Chinese cohort. As a blood-based biomarker, pSMI may serve as a practical screening tool and prognostic marker, facilitating timely interventions such as nutritional support and resistance exercise.

## Introduction

Sarcopenia, characterized by progressive loss of skeletal muscle mass, strength, and physical performance, is an increasingly recognized public health issue among older adults. It is associated with numerous adverse outcomes, including frailty, falls, disability, fractures, institutionalization, and increased mortality (1–3). As the global population continues to age, the prevalence of sarcopenia among individuals aged 60 years and older is estimated to range between 10 and 27%, depending on the diagnostic criteria used (4). This proportion is projected to rise in the coming decades, highlighting the urgent need for effective screening tools and early interventions (5, 6).

Current sarcopenia diagnosis typically relies on advanced imaging techniques such as dual-energy X-ray absorptiometry (DXA), computed tomography (CT), or bioelectrical impedance analysis (BIA), which provide accurate assessments of muscle mass and function (1, 7, 8). However, these methods are costly, time-consuming, and often inaccessible in community or primary care settings, particularly in low-resource environments. These limitations underscore the need for simple, scalable, and affordable approaches to identify individuals at risk.

In this context, blood-based biomarkers have gained attention as potential surrogates for muscle mass estimation. Among them, serum creatinine and cystatin C are commonly used for renal function assessment, but also reflect aspects of muscle metabolism: creatinine is derived from muscle breakdown, while cystatin C is independent of muscle mass (9). Based on this rationale, several composite indices have been developed using the creatinine-to-cystatin C ratio, such as the Cr/CysC ratio, Sarcopenia Index, and more recently, the pSMI, which may serve as a non-invasive proxy for estimating muscle mass and identifying individuals at risk of sarcopenia (10–14).

Recent studies have demonstrated the clinical relevance of pSMI. Jang et al. (15) found that pSMI had the strongest correlation with DXA-defined sarcopenia and the highest discriminative ability among several indices in Korean community-dwelling older adults. Matsuzawa et al. (16) observed that lower pSMI was significantly associated with sarcopenia defined by BIA in community-dwelling older adults in Japan. In China, Ning et al. (17) reported that lower baseline pSMI predicted a significantly higher risk of incident sarcopenia over 4 years in a large prospective study using CHARLS data, reinforcing its potential as an early indicator of muscle decline. While these studies confirm the diagnostic relevance of pSMI in diverse populations and clinical settings, they primarily focused on cross-sectional associations or short-term outcomes. To date, few studies have examined the prognostic utility of pSMI for long-term outcomes such as mortality in large, community-based aging cohorts.

Therefore, the present study aimed to evaluate the association of pSMI with sarcopenia prevalence and five-year all-cause mortality in a nationally representative sample of older Chinese adults. Although prior studies have established pSMI’s utility for sarcopenia diagnosis, evidence regarding its independent prognostic value for long-term mortality in large-scale representative aging populations remains limited. By leveraging routine blood biomarkers and validated estimation methods, we sought to explore pSMI’s potential as a scalable tool for early risk stratification in population aging.

## Methods

### Study population

This study utilized data from the 2015 wave of the China Health and Retirement Longitudinal Study (CHARLS), a nationally representative longitudinal survey of middle-aged and older adults in China. CHARLS employs a multistage probability sampling strategy covering 150 counties and 450 communities across 28 provinces. The study protocol was approved by the Peking University Ethical Review Committee, and all participants provided informed consent. Of 9,845 participants aged ≥60 years, we excluded 3,181 without blood samples, 234 lacking pSMI data, 25 without height, 68 without sarcopenia assessments, and 59 with incomplete covariates, leaving 6,278 for sarcopenia analysis. For mortality, 296 lost to follow-up were excluded, yielding 5,982 participants. As detailed in Supplementary Table S1, excluded men and women were significantly older, more likely to be single (especially women), had lower educational attainment, and exhibited poorer physical function (e.g., lower grip strength, higher proportion with abnormal physical performance). Excluded men were less likely to smoke or drink, while excluded women had lower weight, height, and appendicular skeletal muscle mass (ASM). Notably, some biomarkers like hs-CRP (in men) and cystatin C (in both sexes) also showed significant differences. All participant data were anonymized to protect privacy, in accordance with ethical guidelines and the requirements of CHARLS.

### pSMI computation

pSMI was calculated using a previously developed sex-specific predictive equation (11). The equation was developed as follows:

- **Men:** pSMI = 4.17–0.012 × age + 1.24 × (serum creatinine/serum cystatin C) – 0.0513 × hemoglobin + 0.0598 × body weight.
- **Women:** pSMI = 3.55–0.00765 × age + 0.852 × (serum creatinine/serum cystatin C) – 0.0627 × hemoglobin + 0.0614 × body weight.

Blood samples were analyzed for creatinine (Jaffe method), cystatin C (turbidimetric immunoassay), hemoglobin (automated analyzers) at laboratories (details in Supplementary file). Body weight was measured to the nearest 0.1 kg using a digital scale.

### Other measurements and covariates

In 2015, trained study personnel gathered comprehensive demographic and health data, adhering to standardized protocols and maintaining rigorous quality control. Demographic variables included age, sex, residential area (urban vs. rural, per China National Bureau of Statistics definitions), marital status (married vs. single, where “single” included never married, divorced, or widowed), and education level. Education level was self-reported and categorized into three groups: primary school or below, middle school, and high school or above. Smoking status was classified as current smoker or never/former smoker. Alcohol consumption was defined as any alcohol intake in the past year (yes/no). Anthropometric measurements included height, and waist circumference, obtained using standard methods. WHtR was calculated as waist circumference divided by height, both in centimeters. Further laboratory assessments included the measurement of low-density lipoprotein (LDL) cholesterol (enzymatic colorimetric), triglycerides (enzymatic colorimetric), and high-sensitivity C-reactive protein (hs-CRP) (immunoturbidimetric assay) (details in Supplementary file). For analysis, triglyceride and CRP values were log-transformed (log-TG, log-CRP) to normalize their distributions. Log-transformation was also applied to serum creatinine (log-Cr) when included as a covariate, to account for residual confounding by renal function not captured by pSMI. The presence of chronic conditions was documented based on self-reported physician diagnoses. These comorbidities included hypertension, diabetes, lung disease, heart disease, stroke, psychiatric illness, dyslipidemia, liver disease, kidney disease, cancer, digestive disease, and arthritis or rheumatic disease. All study procedures adhered to ethical standards, and participants provided informed consent prior to data collection.

### Sarcopenia definition

Baseline sarcopenia was classified according to the 2019 consensus definition from the Asian Working Group for Sarcopenia (AWGS 2019) (1). In brief, AWGS 2019 defines sarcopenia as low muscle mass combined with either low muscle strength or low physical performance. Accordingly, we assessed three components in 2015: muscle mass, muscle strength, and physical performance.

- **Muscle mass:** Muscle mass was not directly measured, but estimated appendicular skeletal muscle mass (ASM) for each participant using a previously validated anthropometric equation specific to Chinese older adults (18, 19). This proxy approach, although limited in diagnostic precision, supports risk stratification in population-based studies. The skeletal muscle index (SMI) was then calculated as ASM divided by height squared (kg/m^2^). Low muscle mass was defined as SMI below the sex-specific 20th percentile of this study population (cut-offs: <6.88 kg/m^2^ for men and <5.09 kg/m^2^ for women).
- **Muscle strength:** Grip strength was measured as an indicator of upper-body muscle strength. Participants squeezed a hand dynamometer twice with each hand; the maximum reading (in kilograms) was recorded. Low grip strength was defined as <28 kg for men or <18 kg for women, per AWGS 2019 criteria (1).
- **Physical performance:** We evaluated usual gait speed, the five-time chair-stand test, and overall physical performance via the short physical performance battery (SPPB). Gait speed was measured twice over a 2.5-m walk, with <1.0 m/s indicating low performance. The chair-stand test recorded the time to rise from a chair five times; taking ≥12 s was considered poor performance. SPPB is a composite score of balance, gait, and chair-stand ability; a score ≤9 was considered low performance (20). Satisfying any one of these criteria indicated low physical performance, consistent with AWGS 2019 thresholds (1).

Using the aforementioned components, participants were categorized as exhibiting sarcopenia if they demonstrated low muscle mass and either low muscle strength or low physical performance (or both). Participants failing to meet these criteria were designated as non-sarcopenic. AWGS further subdivides severity (e.g., “severe sarcopenia” if all three criteria are met), but for simplicity, all individuals meeting the sarcopenia definition (with or without severe criteria) were treated as one group.

### Mortality ascertainment

Mortality status was primarily ascertained through proxy reports from family members during the 2018 and 2020 follow-up interviews. All-cause mortality was defined as death from any cause occurring between the baseline interview in 2015 and the date of last follow-up in 2020. Survival time was calculated from the date of the baseline interview to the reported date of death.

### Statistical analysis

We categorized the pSMI into sex-specific quartiles for analyses and also examined it as a continuous variable. Proportions for categorical variables and mean values for continuous variables were used to describe the characteristics by sex-specific pSMI quartiles. Differences across pSMI quartiles were examined with linear regression for continuous variables and Cochran–Armitage trend tests or chi-square tests for categorical variables. Polychotomous logistic regression was used to assess cross-sectional associations between pSMI quartiles and sarcopenia. Models included (0) an unadjusted model; (1) model 1: adjusted for age and WHtR; (2) model 2: further adjusting from model 1 for area, marriage, education level, smoking, drinking, triglycerides, high-sensitivity C-reactive protein (hs-CRP), serum creatinine, hypertension, diabetes, lung disease, cancer, heart disease, stroke, psychiatric disease, liver disease, kidney disease, digestive disease, joint disease. We also examined crude associations with each of the five sarcopenia components using logistic regression and calculated the *p* value for trend across pSMI quartiles. This trend test is the linear effect of pSMI quartiles, treating the quartiles as an ordinal variable and assuming each increase in quartile carries the same incremental effect.

For the longitudinal analysis of mortality, Cox proportional hazards regression models were used to estimate hazard ratios (HRs) for all-cause mortality across sex-specific quartiles of pSMI, using Q1 (lowest quartile) as the reference. The models were adjusted sequentially, as described above. A sensitivity analysis was performed by additionally adjusting for baseline sarcopenia status to examine whether the association between pSMI and mortality was independent of clinically defined sarcopenia. The proportional hazards assumption was assessed graphically and was not violated. To visualize survival differences by pSMI quartiles, sex-stratified Kaplan–Meier survival curves were generated, and the log-rank test was applied to assess statistical significance. Furthermore, to explore potential non-linear associations between continuous pSMI and mortality risk, we fitted a Cox model with restricted cubic splines, using the sex-specific median pSMI as the reference point.

All statistical analyses were conducted using Stata version 18.0 (StataCorp, College Station, TX, USA) and R version 4.4.3 (R Foundation for Statistical Computing, Vienna, Austria). A two-sided *p*-value < 0.05 was considered statistically significant.

## Results

### Baseline characteristics

Baseline characteristics stratified by sex-specific quartiles of pSMI are presented in Table 1. For a comprehensive overview of all baseline variables and their distributions, please refer to Supplementary Table 1. Individuals in the highest pSMI quartile (Q4) were generally younger, taller, and had greater body weight and waist-to-height ratio (WHtR) compared to those in the lowest quartile (Q1) for both sexes (all *p* for trend < 0.001). Additionally, higher pSMI was associated with urban residence, being married, and higher educational attainment.

**Table 1:** Baseline characteristics of older Chinese adults by sex-specific quartiles of pSMI.

Characteristics	Overall	Men, *N* = 3,130					Women, *N* = 3,148				
		pSMI Quartiles					pSMI Quartiles				
		1 *N* = 783	2 *N* = 783	3 *N* = 782	4 *N* = 782	*p* for trend	1 *N* = 787	2 *N* = 787	3 *N* = 787	4 *N* = 787	*p* for trend
pSMI	6.91 (0.94)	6.61 (0.29)	7.23 (0.14)	7.72 (0.15)	8.52 (0.48)	<0.001	5.50 (0.28)	6.07 (0.12)	6.48 (0.13)	7.16 (0.41)	<0.001
Age, years	67.93 (6.45)	71.74 (7.05)	68.28 (6.27)	66.85 (5.56)	65.57 (4.80)	<0.001	71.24 (7.52)	67.81 (6.11)	66.43 (5.47)	65.50 (5.05)	<0.001
Area						<0.001					<0.001
Urban Community	2,361 (37.6%)	184 (23.5%)	254 (32.4%)	324 (41.4%)	383 (49.0%)		221 (28.1%)	278 (35.3%)	333 (42.3%)	384 (48.8%)	
Rural Village	3,917 (62.4%)	599 (76.5%)	529 (67.6%)	458 (58.6%)	399 (51.0%)		566 (71.9%)	509 (64.7%)	454 (57.7%)	403 (51.2%)	
Marriage						<0.001					<0.001
Single	1,336 (21.3%)	160 (20.4%)	133 (17.0%)	99 (12.7%)	79 (10.1%)		276 (35.1%)	241 (30.6%)	183 (23.3%)	165 (21.0%)	
Married	4,942 (78.7%)	623 (79.6%)	650 (83.0%)	683 (87.3%)	703 (89.9%)		511 (64.9%)	546 (69.4%)	604 (76.7%)	622 (79.0%)	
Education level						<0.001					<0.001
Primary and below	5,033 (80.2%)	643 (82.1%)	598 (76.4%)	526 (67.3%)	468 (59.8%)		737 (93.6%)	714 (90.7%)	692 (87.9%)	655 (83.2%)	
Middle	854 (13.6%)	100 (12.8%)	131 (16.7%)	174 (22.3%)	202 (25.8%)		36 (4.6%)	49 (6.2%)	67 (8.5%)	95 (12.1%)	
High and above	391 (6.2%)	40 (5.1%)	54 (6.9%)	82 (10.5%)	112 (14.3%)		14 (1.8%)	24 (3.0%)	28 (3.6%)	37 (4.7%)	
Smoking	1760 (28.0%)	463 (59.1%)	416 (53.1%)	378 (48.3%)	306 (39.1%)	<0.001	71 (9.0%)	53 (6.7%)	42 (5.3%)	31 (3.9%)	<0.001
Drinking	2,955 (47.1%)	534 (68.2%)	563 (71.9%)	556 (71.1%)	583 (74.6%)	0.011	193 (24.5%)	201 (25.5%)	161 (20.5%)	164 (20.8%)	0.016
Weight, kg	57.85 (11.09)	48.78 (4.92)	56.66 (4.30)	64.06 (4.67)	74.27 (8.36)	<0.001	42.91 (4.49)	51.31 (3.29)	57.51 (3.64)	67.40 (7.49)	<0.001
Height, cm	156.87 (8.68)	157.95 (5.99)	161.70 (5.70)	164.22 (5.64)	167.10 (5.83)	<0.001	146.76 (6.09)	150.08 (5.28)	152.19 (4.75)	155.13 (5.02)	<0.001
WHtR	0.55 (0.07)	0.48 (0.05)	0.51 (0.05)	0.54 (0.05)	0.57 (0.05)	<0.001	0.53 (0.06)	0.57 (0.06)	0.59 (0.05)	0.63 (0.06)	<0.001
Hemoglobin, g/dL	13.58 (1.83)	13.92 (1.90)	14.18 (1.81)	14.44 (1.66)	14.60 (1.64)	<0.001	12.73 (1.88)	12.89 (1.54)	12.95 (1.48)	12.93 (1.45)	0.010
LDL cholesterol, mg/dL	103.68 (29.02)	94.96 (26.35)	98.79 (29.33)	100.58 (27.33)	100.46 (28.43)	<0.001	106.97 (29.15)	109.21 (28.61)	108.98 (28.84)	109.35 (30.19)	0.138
Serum Creatinine, mg/dL	0.79 (0.68, 0.93)	0.85 (0.75, 0.98)	0.88 (0.77, 1.00)	0.89 (0.80, 1.00)	0.94 (0.83, 1.07)	<0.001	0.69 (0.60, 0.78)	0.69 (0.62, 0.78)	0.70 (0.62, 0.79)	0.71 (0.64, 0.83)	<0.001
Triglycerides, mg/dL	111.50 (81.42, 162.83)	84.96 (67.26, 114.16)	90.27 (69.91, 127.43)	107.96 (79.65, 155.75)	125.66 (88.50, 184.07)	<0.001	102.65 (76.99, 145.13)	119.47 (89.38, 174.34)	134.51 (96.46, 190.27)	145.13 (104.42, 199.12)	<0.001
hs-CRP, mg/L	1.50 (0.80, 2.80)	1.40 (0.70, 3.10)	1.20 (0.70, 2.40)	1.40 (0.80, 2.70)	1.65 (0.90, 3.00)	0.013	1.20 (0.60, 2.50)	1.40 (0.80, 2.40)	1.60 (0.90, 2.80)	2.00 (1.10, 3.40)	<0.001
Hypertension	2,652 (42.2%)	226 (28.9%)	264 (33.7%)	327 (41.8%)	420 (53.7%)	<0.001	251 (31.9%)	313 (39.8%)	383 (48.7%)	468 (59.5%)	<0.001
Diabetes	774 (12.3%)	48 (6.1%)	52 (6.6%)	97 (12.4%)	123 (15.7%)	<0.001	68 (8.6%)	107 (13.6%)	126 (16.0%)	153 (19.4%)	<0.001
Lung disease	1,066 (17.0%)	221 (28.2%)	163 (20.8%)	131 (16.8%)	119 (15.2%)	<0.001	148 (18.8%)	100 (12.7%)	89 (11.3%)	95 (12.1%)	<0.001
Heart disease	1,422 (22.7%)	109 (13.9%)	133 (17.0%)	162 (20.7%)	208 (26.6%)	<0.001	165 (21.0%)	172 (21.9%)	212 (26.9%)	261 (33.2%)	<0.001
Stroke	306 (4.9%)	39 (5.0%)	45 (5.7%)	40 (5.1%)	53 (6.8%)	0.198	28 (3.6%)	28 (3.6%)	34 (4.3%)	39 (5.0%)	0.117
Psychiatric disease	143 (2.3%)	14 (1.8%)	10 (1.3%)	11 (1.4%)	14 (1.8%)	0.945	21 (2.7%)	26 (3.3%)	27 (3.4%)	20 (2.5%)	0.925
Dyslipidemia	1,354 (21.6%)	66 (8.4%)	105 (13.4%)	177 (22.6%)	253 (32.4%)	<0.001	98 (12.5%)	157 (19.9%)	223 (28.3%)	275 (34.9%)	<0.001
Liver disease	416 (6.6%)	48 (6.1%)	53 (6.8%)	56 (7.2%)	68 (8.7%)	0.050	43 (5.5%)	44 (5.6%)	47 (6.0%)	57 (7.2%)	0.133
Kidney disease	664 (10.6%)	86 (11.0%)	108 (13.8%)	105 (13.4%)	94 (12.0%)	0.604	56 (7.1%)	58 (7.4%)	70 (8.9%)	87 (11.1%)	0.003
Cancer	110 (1.8%)	8 (1.0%)	12 (1.5%)	8 (1.0%)	16 (2.0%)	0.173	13 (1.7%)	14 (1.8%)	19 (2.4%)	20 (2.5%)	0.148
Digestive disease	1981 (31.6%)	246 (31.4%)	221 (28.2%)	209 (26.7%)	185 (23.7%)	<0.001	306 (38.9%)	282 (35.8%)	273 (34.7%)	259 (32.9%)	0.013
Joint disease	2,950 (47.0%)	346 (44.2%)	334 (42.7%)	294 (37.6%)	292 (37.3%)	0.001	414 (52.6%)	432 (54.9%)	403 (51.2%)	435 (55.3%)	0.587

Mean (SD) or n (%) shown in the table. ACR, hs-CRP and Cr show median [IQR].

*p* value for trend: (1) Linear regression for trend analysis of normally distributed variables; (2) Linear regression after log-transformation for TG, CRP, Cr; (3) Cochran-Armitage trend test for binary variables; (4) Chi-square test for categorical variables with >2 levels. CHARLS study: China health and retirement longitudinal study; pSMI: predictive skeletal muscle mass index; WHtR: waist-to-height ratio.

Sex-specific differences emerged in metabolic and inflammatory profiles. Among men, median triglyceride levels increased from 84.96 [67.26–114.16] mg/dL in Q1 to 125.66 [88.50–184.07] mg/dL in Q4 (*p* for trend < 0.001). High-sensitivity C-reactive protein (hs-CRP) levels showed a U-shaped pattern, dipping in Q2 (1.20 [0.70–2.40] mg/L) and rising in Q4 (1.65 [0.90–3.00] mg/L) compared to Q1 (1.40 [0.70–3.10] mg/L; p for trend = 0.013). In women, both triglyceride and hs-CRP levels increased progressively across pSMI quartiles, suggesting greater metabolic and inflammatory burden at higher pSMI levels (TG: Q1 = 102.65 [76.99–145.13] to Q4 = 145.13 [104.42–199.12] mg/dL; hs-CRP: Q1 = 1.20 [0.60–2.50] to Q4 = 2.00 [1.10–3.40] mg/L; both *p* < 0.001).

Prevalence of chronic diseases also varied by pSMI quartile. In men, lung disease and digestive disorders were more common in Q1 (*p* < 0.001), whereas cardiometabolic conditions such as hypertension, diabetes, and heart disease became increasingly prevalent with rising pSMI. Similar patterns were observed in women, with cardiometabolic risk (including hypertension, diabetes, and heart disease) increasing and respiratory conditions declining across quartiles. Collectively, these findings suggest distinct health profiles across the pSMI spectrum: individuals in Q1 exhibited greater multi-system vulnerability, while those in Q4 had elevated cardiometabolic and inflammatory risk, especially among women. These health differences may partially reflect underlying socioeconomic disparities, as higher pSMI was also linked to urban residence, marital status, and higher education.

### Sarcopenia prevalence and association with pSMI

A striking inverse gradient in sarcopenia prevalence was observed across pSMI quartiles for both sexes (Table 2). Over half of the participants in Q1 met the criteria for sarcopenia (men: 55.3%; women: 66.6%), compared to only 0.4% in Q4 for both sexes. Intermediate quartiles (Q2–Q3) showed progressively lower prevalence, indicating a clear dose–response relationship (p for trend < 0.001).

**Table 2:** Sex-specific associations of pSMI quartiles with sarcopenia and its components at baseline.

Sarcopenia and sarcopenia components	Men, *N* = 3,130					Women, *N* = 3,148				
	pSMI quartile					pSMI quartile				
	Q1	Q2	Q3	Q4	p for trend	Q1	Q2	Q3	Q4	p for trend
*N*	783	783	782	782		787	787	787	787	
Distributions, *n* (%)										
Sarcopenia	433 (55.3%)	84 (10.7%)	9 (1.2%)	3 (0.4%)	<0.001	524 (66.6%)	34 (4.3%)	7 (0.9%)	3 (0.4%)	<0.001
Robust	350 (44.7%)	699 (89.3%)	773 (98.8%)	779 (99.6%)	<0.001	263 (33.4%)	753 (95.7%)	780 (99.1%)	784 (99.6%)	<0.001
Sarcopenia components										
Low muscle mass	491 (62.7%)	118 (15.1%)	11 (1.4%)	7 (0.9%)	<0.001	576 (73.2%)	38 (4.8%)	8 (1.0%)	3 (0.4%)	<0.001
Weak grip strength	291 (37.2%)	140 (17.9%)	99 (12.7%)	65 (8.3%)	<0.001	234 (29.7%)	119 (15.1%)	96 (12.2%)	86 (10.9%)	<0.001
Slow walking speed	621 (81.2%)	544 (71.0%)	515 (67.1%)	499 (65.7%)	<0.001	666 (86.4%)	638 (82.9%)	617 (79.9%)	611 (80.5%)	<0.001
Low chair stand	165 (22.1%)	148 (19.3%)	135 (17.7%)	143 (19.0%)	0.092	250 (34.1%)	217 (28.8%)	190 (24.9%)	225 (29.9%)	0.027
Low physical performance	144 (19.9)	100 (13.4%)	89 (11.9%)	69 (9.5%)	<0.001	240 (33.8%)	165 (22.5%)	141 (18.9%)	153 (21.1%)	<0.001

Cross-sectional polychotomous logistic regression of sarcopenia as an outcome (compared with robust); OR [95% CIs]						
	Q1	Q2	Q3–Q4	Q1	Q2	Q3–Q4
Unadjusted	1 (ref)	0.097 [0.074–0.127]***	0.006 [0.004–0.011]***	1 (ref)	0.023 [0.016–0.033]***	0.003 [0.0 02to 0.006]***
Model 1	1 (ref)	0.142 [0.104–0.192]***	0.030 [0.016–0.056]***	1 (ref)	0.034 [0.023–0.050]***	0.011 [0.005–0.020]***
Model 2	1 (ref)	0.133 [0.096–0.184]***	0.027 [0.014–0.053]***	1 (ref)	0.026 [0.017–0.039]***	0.006 [0.003–0.012]***

Model 1 adjustment includes Age, WHtR; Model 2 adjustment includes Age, WHtR, Area, Marriage, Education level, Smoking, Lung disease, Drinking, Hypertension, Diabetes, Cancer, Heart disease, Stroke, Psychiatric disease, Liver disease, Kidney disease, Digestive disease, Joint disease, logTG, logCRP, logCr. CHARLS study: China health and retirement longitudinal study; pSMI: predicted Skeletal Muscle Index; OR, odds ratio; CI, confidence interval; WHtR: waist-to-height ratio.

*p < 0.05, **p < 0.01, ***p < 0.001.

Logistic regression confirmed a strong inverse association between pSMI and sarcopenia odds. In fully adjusted models, individuals in Q3–Q4 had significantly lower odds of sarcopenia compared to Q1 (men: OR = 0.027, 95% CI: 0.014–0.053; women: OR = 0.006, 95% CI: 0.003–0.012). Even those in Q2 had markedly reduced odds (men: OR = 0.133; women: OR = 0.026; both *p* < 0.001).

Analyses of sarcopenia components demonstrated robust trends across pSMI quartiles. Among men in Q1, 62.7% had low muscle mass, 37.2% had weak grip strength, 81.2% had slow gait, 22.1% failed the chair-stand test, and 19.9% had poor SPPB scores. These proportions declined significantly in Q4 (0.9, 8.3, 65.7, 19.0, and 9.5%, respectively), though the chair-stand trend was not statistically significant (*p* = 0.092).

In women, impairments were more prevalent and consistently declined across pSMI quartiles. For Q1 vs. Q4: low muscle mass (73.2% vs. 0.4%), weak grip strength (29.7% vs. 10.9%), slow gait (86.4% vs. 80.5%), poor chair-stand (34.1% vs. 29.9%), and low SPPB score (33.8% vs. 21.1%). All trends were statistically significant (p for trend < 0.001), except for the chair-stand test (*p* = 0.027). These consistent associations support the validity of pSMI as a marker of muscle quality and functional decline.

### pSMI and mortality

**Figure 1:**
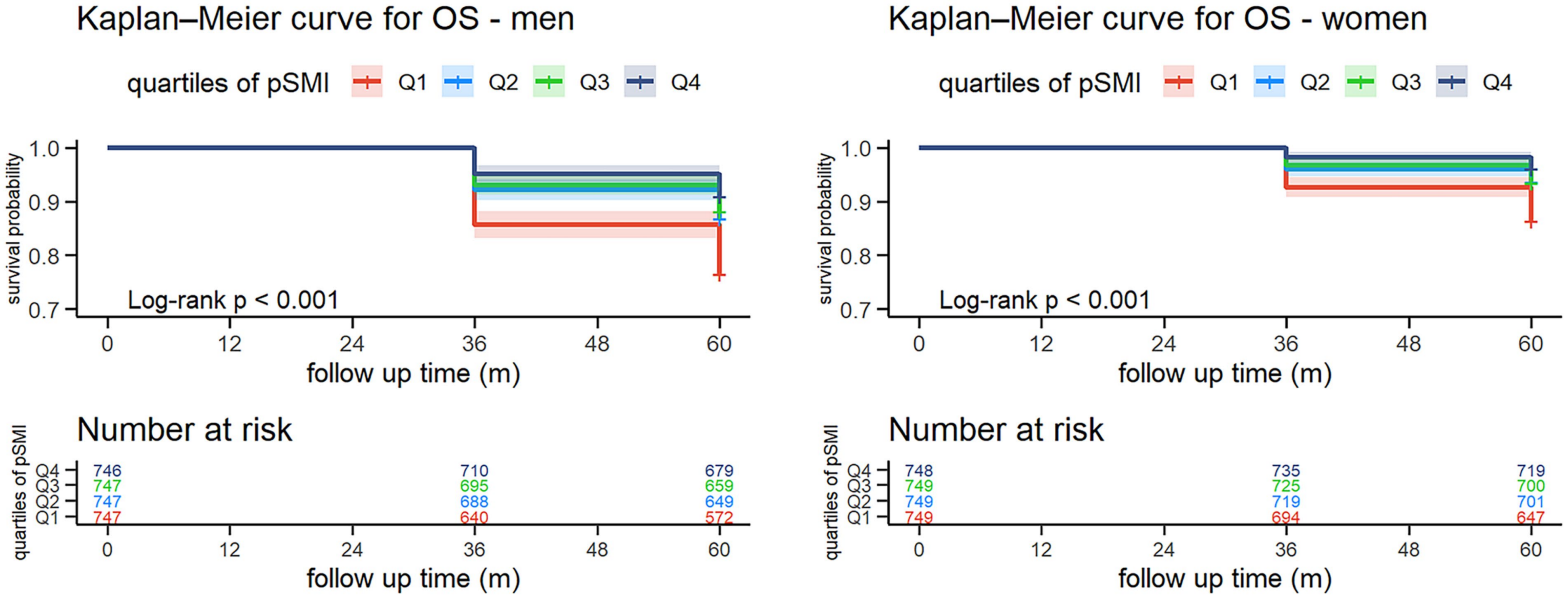
Kaplan–Meier survival curves for all-cause mortality by sex-specific quartiles of pSMI. Participants with higher baseline pSMI had significantly greater 5-year survival probabilities compared to those in the lowest quartile (log-rank test, *p* < 0.001 for both sexes). Numbers at risk are displayed below each curve.

During the five-year follow-up (2015–2020), 656 deaths occurred (428 men, 228 women) (Table 3). Mortality declined progressively across pSMI quartiles. In men, it decreased from 23.4% in Q1 to 9.0% in Q4; in women, from 13.6 to 3.9%. Kaplan–Meier curves demonstrated significantly higher survival in higher pSMI quartiles (log-rank *p* < 0.001 for both sexes) (Figure 1).

**Table 3:** Cox regression models assessing all-cause mortality risk by pSMI quartiles in older Chinese adults.

	pSMI quartiles	Q1	Q2	Q3	Q4
Men, *N* = 2,987 (Events = 428)					
Events (%)		175 (23.4%)	98 (13.1%)	88 (11.8%)	67 (9%)
HR [95% CI]	Unadjusted	1 (ref)	0.53 [0.41–0.68]***	0.47 [0.36–0.61]***	0.35 [0.27–0.47]***
	Model 1	1 (ref)	0.64 [0.5–0.83]***	0.62 [0.46–0.83]**	0.48 [0.33–0.69]***
	Model 2	1 (ref)	0.67 [0.51–0.87]**	0.64 [0.47–0.88]**	0.47 [0.32–0.70]***
	Sensitivity with sarcopenia	1 (ref)	0.81 [0.60–1.08]	0.76 [0.54–1.08]	0.57 [0.38–0.87]**
Women, *N* = 2,995 (Events = 228)					
Events (%)		102 (13.6%)	48 (6.4%)	49 (6.5%)	29 (3.9%)
HR [95% CI]	Unadjusted	1 (ref)	0.45 [0.32–0.64]***	0.46 [0.33–0.65]***	0.27 [0.18–0.41]***
	Model 1	1 (ref)	0.67 [0.46–0.97]*	0.81 [0.55–1.2]	0.50 [0.31–0.83]**
	Model 2	1 (ref)	0.64 [0.44–0.93]*	0.72 [0.48–1.09]	0.38 [0.22–0.65]***
	Sensitivity with sarcopenia	1 (ref)	0.71 [0.44–1.15]	0.81 [0.49–1.35]	0.40 [0.22–0.75]**

Model 1: Age, WHtR; Model 2: Age, WHtR, Area, Marriage, Education level, Smoking, Lung disease, Drinking, Hypertension, Diabetes, Cancer, Heart disease, Stroke, Psychiatric disease, Liver disease, Kidney disease, Digestive disease, logTG, logCRP and logCr. Sensitivity with sarcopenia: Age, WHtR, Area, Marriage, Education level, Smoking, Lung disease, Drinking, Marriage, Hypertension, Diabetes, Cancer, Heart disease, Stroke, Psychiatric disease, Liver disease, Kidney disease, Digestive disease, Joint disease, logTG, logCRP, logCr and sarcopenia status. pSMI: predicted Skeletal Muscle Index; HR: Hazard Ratio; CI: confidence interval; WHtR: waist-to-height ratio.

*p < 0.05, **p < 0.01, ***p < 0.001.

**Figure 2:**
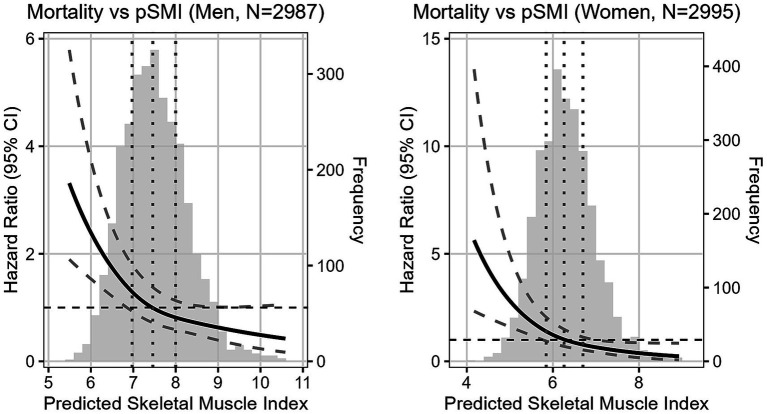
Adjusted HR of mortality following baseline continuous pSMI in men and women. Reference point is the median pSMI. Vertical dotted lines represent quartiles. Adjusted for Age, WHtR, Area, Marriage, Education level, Smoking, Lung disease, Drinking, Marriage, Hypertension, Diabetes, Cancer, Heart disease, Stroke, Psychiatric disease, Liver disease, Kidney disease, Digestive disease, logTG, logCRP, and logCr.

In Cox models, higher pSMI was associated with reduced all-cause mortality. Fully adjusted hazard ratios for Q4 vs. Q1 were 0.47 (95% CI: 0.32–0.70, *p* < 0.001) in men and 0.38 (95% CI: 0.22–0.65, *p* < 0.001) in women. Sensitivity analysis further confirmed the independence of this association from sarcopenia status (HR = 0.57 in men, 0.40 in women). Furthermore, restricted cubic spline analysis (Figure 2) confirmed a non-linear, dose-dependent association between continuous pSMI and mortality risk, with lower pSMI values associated with a steep increase in hazard, particularly below the median.

## Discussion

In this large prospective cohort of 5,982 community-dwelling older Chinese adults, we found a strong inverse association between pSMI and both sarcopenia prevalence and five-year all-cause mortality. Sarcopenia prevalence showed a marked decline from 55.3 to 0.4% in men and from 66.6 to 0.4% in women across increasing pSMI quartiles. Similarly, mortality declined from 23.4 to 9.0% in men and from 13.6 to 3.9% in women. These associations remained significant after adjusting for multiple covariates, and dose–response relationships were confirmed using Kaplan–Meier and spline analyses, highlighting especially steep risk at the lowest pSMI levels.

Beyond its biological relevance, our findings show that higher pSMI was consistently associated with favorable socioeconomic factors such as urban residence, marital status, and higher educational attainment, as observed in our baseline data. These patterns suggest that social determinants may support muscle preservation by promoting healthier behaviors, better nutrition, and improved access to healthcare—a relationship broadly supported by existing evidence linking socioeconomic status to a range of health outcomes (21). This highlights the importance of considering such factors as potential confounders or mediators when assessing aging-related risks and interpreting the role of pSMI in diverse populations.

Sarcopenia is a well-established risk factor for frailty, functional decline, and mortality in older adults, yet it remains underdiagnosed due to the limited availability of specialized assessment tools (1, 7, 22–24). The use of pSMI, calculated from routine creatinine and cystatin C levels, offers a practical alternative (11). While prior studies by Jang et al. (15) highlighted pSMI’s strong correlation with DXA-defined sarcopenia in Korean older adults, and Matsuzawa et al. (16) demonstrated its relevance in Japanese older adults, and Ning et al. (17) prospectively linked it to incident sarcopenia in a broader Chinese cohort including adults aged 45 and older (also utilizing CHARLS data), respectively, our study uniquely focuses on Chinese older adults (≥60 years), using a population-specific pSMI equation. We not only confirmed its association with sarcopenia prevalence, but, to our knowledge, also provide the first large-scale prospective validation of pSMI as an independent predictor of five-year all-cause mortality, even after adjusting for baseline sarcopenia. The association with poorer physical performance—grip strength, gait speed, and SPPB tests—further supports its relevance as an integrative marker of overall health status in aging populations.

Interestingly, while low pSMI indicates vulnerability, higher pSMI was not uniformly beneficial. Participants in the highest quartiles had higher triglyceride levels, WHtR, and greater prevalence of hypertension and diabetes. Furthermore, a significant trend in the prevalence of heart disease was also observed across pSMI quartiles in both men and women, reinforcing the notion of a complex interplay between muscle mass and overall cardiovascular health indicators. These patterns suggest a mixed phenotype of high muscle mass accompanied by increased adiposity. This was especially evident in women, where hs-CRP—a marker of systemic inflammation—increased consistently across pSMI quartiles. In contrast, men showed a less consistent trend. These sex differences likely reflect hormonal and metabolic influences, including greater visceral fat accumulation in postmenopausal women (25)—a key factor as visceral fat significantly contributes to systemic inflammation (26)—and the anti-inflammatory effects of testosterone in men (27).

Our results are consistent with those of Liu et al. (23), who reported that skeletal muscle mass is positively correlated with absolute fat mass. In our study, high pSMI combined with elevated WHtR may indicate a condition where both muscle and fat increase. Although muscle mass may reduce sarcopenia risk, excess fat—particularly visceral—may drive cardiometabolic complications via mechanisms like insulin resistance and chronic low-grade inflammation (28). These findings emphasize the need to assess muscle and fat jointly, rather than in isolation.

At the other end of the spectrum, participants with low pSMI had a higher burden of respiratory and gastrointestinal illnesses (17), consistent with broader physiological decline. Surprisingly, hs-CRP levels were not elevated in this group. This may be explained by protein-energy malnutrition, which can impair hepatic synthesis of inflammatory proteins (29), and reduced muscle tissue, which contributes to basal cytokine production (30). Additionally, impaired renal clearance, which influences cystatin C and CRP levels, may complicate interpretation (9). These findings suggest that hs-CRP alone may not reliably reflect inflammatory status in sarcopenic older adults with severe muscle loss and possible malnutrition. Assessing additional biomarkers, such as IL-6 or TNF-α, may provide a more accurate picture of systemic inflammation in this context (31, 32).

Taken together, our findings affirm pSMI as a clinically useful marker across a broad spectrum of risk. Low pSMI reflects frailty and poor survival prospects, while high pSMI may mask hidden metabolic risk, especially in women. Therefore, interpreting pSMI requires a context-sensitive approach, incorporating anthropometric indicators, inflammatory markers, and functional assessments to fully capture an individual’s health trajectory.

From a clinical perspective, pSMI offers a scalable and accessible means to screen for sarcopenia and mortality risk in primary care, enabling personalized intervention strategies. Individuals with low pSMI should be prioritized for early intervention, including nutritional support with 1.2–2.0 g/kg/day of protein and regular resistance training, as recommended by current guidelines (1, 7, 33, 34). For those with high pSMI and coexisting metabolic risks—particularly women with elevated WHtR or hs-CRP—additional strategies focused on fat reduction and cardiometabolic health are recommended. Men may also benefit from targeted interventions based on individual risk profiles.

## Strengths

Our study has several notable strengths. First, we utilized a large, nationally representative cohort of older adults, enhancing the generalizability of our findings within the Chinese population. Second, data collection was guided by standardized protocols, and statistical analyses were conducted using robust models that accounted for a comprehensive range of demographic, behavioral, clinical, and biochemical covariates. Third, the application of the AWGS 2019 criteria for sarcopenia diagnosis allows for direct comparability with other studies conducted in Asian populations. Finally, the clear dose–response patterns observed across pSMI quartiles, and the consistency of results after adjusting for sarcopenia status, lend support to the validity of pSMI as a meaningful predictor of adverse outcomes.

## Limitations

Nonetheless, several limitations should be acknowledged. First, a large proportion of participants were excluded due to missing data on blood biomarkers or sarcopenia assessments. While we compared characteristics of included versus excluded participants (Supplementary Table S1) and observed that excluded individuals were older and frailer, which may indicate a missing at random (MAR) mechanism conditional on age and health status, the possibility of non-random missingness cannot be ruled out. Given that both pSMI and mortality risk are closely related to frailty, the missing data mechanism may be closer to MNAR, potentially introducing selection bias and underestimating the associations observed. Future analyses employing advanced statistical methods, such as multiple imputation or inverse probability weighting, are planned to rigorously account for potential selection bias and improve generalizability. Second, as an observational study, we cannot infer causality. To rigorously establish causal relationships, future studies should ideally employ longitudinal interventional designs or incorporate causal inference methods such as Mendelian randomization, instrumental variable analysis, or longitudinal mediation analyses. Third, muscle mass was estimated using a validated anthropometric formula (18) rather than imaging modalities such as DXA, potentially introducing measurement error. Fourth, we did not analyze cause-specific mortality; it remains unclear whether pSMI is more predictive of certain outcomes (e.g., cardiovascular vs. infectious deaths). Fifth, although the five-year follow-up period is informative, longer-term prognostic trajectories may not be fully captured. Lastly, while pSMI was derived from a validated equation (11), its prognostic performance relative to direct muscle mass assessments remains to be established in more diverse and heterogeneous populations.

## Future directions

Future studies should address these limitations through methodological refinements and broader validation efforts. For instance, the use of multiple imputation, inverse probability weighting, or other statistical correction methods may help reduce selection bias and enhance the representativeness of study samples. Validation in independent cohorts—particularly those with complete data and higher proportions of frail or institutionalized older adults—will be essential for confirming the generalizability of our findings. Additionally, research exploring the role of sex hormones and adipokine profiles (e.g., adiponectin, leptin) in modulating the interplay between pSMI, adiposity, and inflammation could provide mechanistic insight into observed sex-specific differences. The use of more sensitive inflammatory markers, such as IL-6 and TNF-α, may improve the characterization of systemic inflammation in low pSMI individuals and clarify the limited discriminatory capacity of hs-CRP in sarcopenia. Exploring these markers could provide essential insights into the mechanisms linking muscle loss, inflammation, and mortality, thereby refining the clinical interpretation of pSMI in diverse older populations. Moreover, ongoing research is specifically underway to establish optimal clinical cutoff values and develop practical risk-scoring models for pSMI. These efforts will significantly enhance its direct clinical utility and facilitate integration into routine practice.

## Conclusion

pSMI is a practical, cost-effective, and informative marker for identifying older adults at increased risk of sarcopenia and mortality. Its ease of calculation and integration into routine blood tests make it especially valuable in aging societies. When used alongside measures of fat distribution, inflammation, and function, pSMI can support more personalized and timely interventions, ultimately helping preserve health and independence in later life.

## Data Availability

Publicly available datasets were analyzed in this study. This data can be found at: the data used in this study are available from the China Health and Retirement Longitudinal Study (CHARLS) repository (http://charls.pku.edu.cn/). Access to the data is subject to approval by the CHARLS research team.
