# Supplementary material for: Predicted skeletal muscle index as a scalable marker for sarcopenia risk and mortality in older Chinese adults
Source: Front Med (Lausanne). 2025 Aug 20;12:1640410. doi: 10.3389/fmed.2025.1640410 (PMC12405267; doi:10.3389/fmed.2025.1640410)
Supplement: Supplementary file 1 [file Table_1.docx]

**Supplementary Materials**

- Supplementary Table S1: Baseline Characteristics by Inclusion Status and Gender
- Supplementary File: Laboratory Protocols for Biomarker Assays

| **Table S1: Baseline Characteristics of Participants by Inclusion Status and Gender** | | | | | | |
| --- | --- | --- | --- | --- | --- | --- |
| **Characteristics** | **Included Men,**  **N=3130** | **Excluded Men,**  **N = 1728** | **p-value** | **Included Women,**  **N = 3148** | **Excluded Women,**  **N = 1839** | **p-value** |
| Age, years | 68.11(6.41) | 69.00 (7.46) | <0.001 | 67.74 (6.48) | 69.98 (8.32) | <0.001 |
| Area |  |  | <0.001 |  |  | <0.001 |
| Rural Village | 1985 (63.4%) | 975 (56.4%) |  | 1932 (61.4%) | 1032 (56.1%) |  |
| Urban Community | 1145 (36.6%) | 753 (43.6%) |  | 1216 (38.6%) | 807 (43.9%) |  |
| Marriage |  |  | 0.005 |  |  | <0.001 |
| Married | 2605 (83.2%) | 1381 (79.9%) |  | 2251 (71.5%) | 1156 (62.9%) |  |
| Single | 525 (16.8%) | 347 (20.1%) |  | 897 (28.5%) | 683 (37.1%) |  |
| Education level |  |  | <0.001 |  |  | <0.001 |
| High and above | 288 (9.2%) | 236 (13.7%) |  | 103 (3.3%) | 101 (5.5%) |  |
| Middle | 607 (19.4%) | 286 (16.6%) |  | 247 (7.8%) | 176 (9.6%) |  |
| Primary and below | 2235 (71.4%) | 1204 (69.8%) |  | 2798 (88.9%) | 1560 (84.9%) |  |
| Missing | 0 | 2 | - | 0 | 2 |  |
| Smoking | 1563 (49.9%) | 760 (44.4%) | <0.001 | 197 (6.3%) | 118 (6.5%) | 0.800 |
| Missing | 0 | 18 |  | 0 | 19 |  |
| Drinking | 1627 (52.0%) | 798 (47.0%) | <0.001 | 442 (14.0%) | 242 (13.4%) | 0.563 |
| Missing | 0 | 29 |  | 0 | 34 |  |
| Weight, kg | 60.94 (11.04) | 59.90 (12.20) | 0.028 | 54.78 (10.24) | 52.80 (12.68) | <0.001 |
| Missing | 0 | 922 |  | 0 | 979 |  |
| Height, cm | 162.74 (6.69) | 161.85 (7.82) | 0.003 | 151.04 (6.12) | 149.49 (10.42) | <0.001 |
| Missing | 0 | 935 |  | 0 | 984 |  |
| WHtR | 0.53 (0.06) | 0.52 (0.09) | 0.777 | 0.58 (0.07) | 0.58 (0.20) | 0.890 |
| Missing | 0 | 939 |  | 0 | 992 |  |
| ASM, kg | 19.87 (2.68) | 19.54 (2.87) | 0.004 | 13.28 (2.47) | 12.67 (3.08) | <0.001 |
| Missing | 0 | 941 |  | 0 | 993 |  |
| SMI, kg/m² | 7.48 (0.69) | 7.45 (1.00) | 0.490 | 5.79 (0.82) | 5.67 (1.63) | 0.035 |
| Missing | 0 | 941 |  | 0 | 993 |  |
| Grip strength, kg | 34.47 (7.86) | 32.52 (8.93) | <0.001 | 23.15 (6.15) | 20.58 (6.87) | <0.001 |
| Missing | 29 | 909 |  | 59 | 980 |  |
| Physical performance |  |  | <0.001 |  |  | <0.001 |
| Abnormal | 402 (13.7%) | 149 (21.7%) |  | 699 (24.0%) | 257 (37.0%) |  |
| Normal | 2540 (86.3%) | 537 (78.3%) |  | 2218 (76.0%) | 438 (63.0%) |  |
| Missing | 188 | 1042 |  | 231 | 1144 |  |
| pSMI | 7.52 (0.76) | 7.54 (1.05) | 0.860 | 6.30 (0.66) | 6.35 (1.21) | 0.711 |
| Missing | 0 | 1658 |  | 0 | 1757 |  |
| Hemoglobin, g/dL | 14.29 (1.78) | 14.17 (2.17) | 0.536 | 12.88 (1.60) | 12.75 (1.95) | 0.429 |
| Missing | 0 | 1588 |  | 0 | 1681 |  |
| Triglycerides, mg/dL | 98.23 （74.34, 144.25） | 102.66 (75.22, 152.21) | 0.399 | 124.78 (91.15, 178.76) | 135.40(92.04, 198.23) | 0.135 |
| Missing | 0 | 1558 |  | 0 | 1657 |  |
| hs-CRP, mg/L | 1.40 (0.80, 2.70) | 1.80 (1.00, 4.10) | 0.002 | 1.50 (0.80, 2.80) | 1.60 (0.90, 3.80) | 0.084 |
| Missing | 0 | 1559 |  | 0 | 1657 |  |
| Serum Creatinine, mg/dL | 0.89 (0.79, 1.02) | 0.87 (0.78, 1.01) | 0.108 | 0.70 (0.62, 0.79) | 0.68 (0.60, 0.82) | 0.227 |
| Missing | 0 | 1558 |  | 0 | 1657 |  |
| Cystatin C, mg/L | 0.92 (0.80, 1.03) | 0.96 (0.83, 1.08) | 0.009 | 0.87 (0.76, 0.98) | 0.91 (0.78, 1.03) | 0.014 |
| Missing | 0 | 1560 |  | 0 | 1659 |  |
| LDL cholesterol, mg/dL | 98.70 (27.96) | 96.23 (27.75) | 0.261 | 108.63 (29.20) | 107.97 (38.24) | 0.819 |
| Missing | 0 | 1559 |  | 0 | 1657 |  |
| Hypertension | 1237 (39.5%) | 621 (38.0%) | 0.331 | 1415 (44.9%) | 783 (44.4%) | 0.727 |
| Missing | 0 | 95 |  | 0 | 75 |  |
| Diabetes | 320 (10.2%) | 175 (10.8%) | 0.593 | 454 (14.4%) | 231 (13.2%) | 0.236 |
| Missing | 0 | 103 |  | 0 | 83 |  |
| Lung disease | 634 (20.3%) | 348 (21.3%) | 0.408 | 432 (13.7%) | 290 (16.5%) | 0.010 |
| Missing | 0 | 96 |  | 0 | 79 |  |
| Heart disease | 612 (19.6%) | 313 (19.2%) | 0.809 | 810 (25.7%) | 462 (26.2%) | 0.724 |
| Missing | 0 | 99 |  | 0 | 78 |  |
| Stroke | 177 (5.7%) | 138 (8.5%) | <0.001 | 129 (4.1%) | 124 (7.0%) | <0.001 |
| Missing | 0 | 101 |  | 0 | 80 |  |
| Liver disease | 225 (7.2%) | 106 (6.5%) | 0.427 | 191 (6.1%) | 102 (5.8%) | 0.769 |
| Missing | 0 | 103 |  | 0 | 85 |  |
| Kidney disease | 393 (12.6%) | 198 (12.2%) | 0.753 | 271 (8.6%) | 175 (10.0%) | 0.124 |
| Missing | 0 | 104 |  | 0 | 84 |  |
| Cance | 44 (1.4%) | 25 (1.5%) | 0.816 | 66 (2.1%) | 40 (2.3%) | 0.750 |
| Missing | 0 | 102 |  | 0 | 84 |  |
| Digestive disease | 861 (27.5%) | 461 (28.2%) | 0.621 | 1120 (35.6%) | 618 (35.2%) | 0.789 |
| Missing | 0 | 95 |  | 0 | 81 |  |
| Joint diseas | 1266 (40.4%) | 669 (40.9%) | 0.777 | 1684 (53.5%) | 959 (54.5%) | 0.536 |
| Missing | 0 | 93 |  | 0 | 78 |  |
| Dyslipidemia | 601 (19.2%) | 283 (17.6%) | 0.197 | 753 (23.9%) | 321 (18.4%) | <0.001 |
| Missing | 0 | 121 |  | 0 | 93 |  |
| Data are presented as mean ± SD for normally distributed variables, median (IQR) for non-normally distributed variables, and n (%) for categorical variables.  p-values: t-tests for normally distributed continuous variables, Wilcoxon rank-sum tests for non-normally distributed continuous variables, and chi-square tests for categorical variables.  pSMI: predictive skeletal muscle mass index; WHtR: waist-to-height ratio; ASM: Appendicular skeletal muscle mass; SMI: Skeletal muscle index. | | | | | | |

**Laboratory Protocols for Blood Sample Collection and Biomarker Assays**

**Blood Sample Collection and Processing**

Venous blood samples were collected during the 2015 wave of the China Health and Retirement Longitudinal Study (CHARLS) between July and October 2015 (with one primary sampling unit [PSU] collected in 2016). Participants were asked to fast overnight prior to blood collection to standardize biomarker measurements. Blood was drawn by trained phlebotomists at local county health centers or designated CHARLS collection sites. Samples were collected into appropriate tubes: ethylenediaminetetraacetic acid (EDTA) tubes for complete blood count (CBC) analysis (including hemoglobin) and serum separator tubes for other biomarkers. After collection, samples were immediately centrifuged at local health centers to separate serum, then frozen and shipped on dry ice to centralized laboratories for analysis. Serum samples were stored at -80°C until assays were performed. A total of 13,420 individuals provided blood samples out of the 21,100 interviewed in Wave 3, yielding a response rate of 63.60% (Table 2 in the CHARLS Blood Data Release Note).

**Complete Blood Count (CBC) Analysis**

CBC analysis, including hemoglobin, hematocrit, white blood cell count, platelet count, and mean corpuscular volume, was performed at local county health centers on the day of collection. Hemoglobin (variable: bl_hgb) was measured in whole blood using automated hematology analyzers available at each center. The specific method and equipment varied by site, but all centers adhered to standardized protocols to ensure consistency. Hemoglobin values are reported in g/dL, with quality control measures implemented at each center to maintain accuracy and precision. The CHARLS dataset does not specify the exact analytical method (e.g., sodium lauryl sulfate [SLS]-hemoglobin method), but all measurements were conducted under routine clinical laboratory standards.

**Serum Biomarker Assays**

Serum assays for creatinine, cystatin C, high-sensitivity C-reactive protein (hs-CRP), triglycerides, and low-density lipoprotein (LDL) cholesterol were conducted at centralized laboratories. The specific methods for each biomarker are described below, based on the variables released in the CHARLS blood dataset (Table 1 in the CHARLS Blood Data Release Note). While the dataset does not specify the exact equipment or calibration standards, all assays were performed following standardized protocols at certified facilities, with quality control measures in place to ensure reliability.

- **Serum Creatinine (bl_crea)**: Creatinine levels were measured in serum using a rate-blanked and compensated Jaffe method, a common enzymatic approach for creatinine quantification. This method minimizes interference from non-creatinine chromogens by incorporating a rate-blanking step and compensation for pseudo-creatinine reactions. Creatinine values are reported in mg/dL.
- **Serum Cystatin C (bl_cysc)**: Cystatin C was quantified in serum using a particle-enhanced turbidimetric immunoassay (PETIA). In this assay, cystatin C in the sample reacts with anti-cystatin C antibodies coated on latex particles, causing agglutination that is measured turbidimetrically. Cystatin C values are reported in mg/L.
- **High-Sensitivity C-Reactive Protein (bl_crp)**: hs-CRP was measured using an immunoturbidimetric assay. The assay involves the reaction of hs-CRP with anti-CRP antibodies, forming antigen-antibody complexes that increase turbidity, which is then quantified. hs-CRP values are reported in mg/L.
- **Triglycerides (bl_tg)**: Triglycerides were measured using an enzymatic colorimetric assay. This method involves enzymatic hydrolysis of triglycerides to glycerol and free fatty acids, followed by a colorimetric reaction to quantify glycerol concentration. Values are reported in mg/dL. Triglyceride values exceeding 500 mg/dL were top-coded to 500 mg/dL (indicated by the variable bl_top_coding_tg).
- **Low-Density Lipoprotein (LDL) Cholesterol (bl_ldl)**: LDL cholesterol was measured using an enzymatic colorimetric assay. This method selectively quantifies LDL cholesterol through enzymatic reactions producing a colored product, measured spectrophotometrically. LDL cholesterol values are reported in mg/dL.

**Quality Assurance**

All assays were conducted at certified laboratories following standardized protocols. Quality control measures were implemented at both local health centers (for CBC) and centralized laboratories (for serum assays), including the use of internal control samples to monitor precision and accuracy. The CHARLS dataset does not provide specific details on calibration standards or coefficients of variation (CVs), but all measurements were performed under routine clinical laboratory standards to ensure reliability. Cross-sectional weights (Blood_weight) were applied to account for non-response in blood collection, as described in the CHARLS Blood Data Release Note.

**Data Availability**

The released variables for each biomarker are listed in Table 1 of the CHARLS Blood Data Release Note, including units and definitions (e.g., bl_hgb for hemoglobin, bl_crea for creatinine). All data are stored in Stata 13 format, and a codebook summarizing variable information is available from the CHARLS repository.
